# Phase inversion-based nanoemulsions of medium chain triglyceride as potential drug delivery system for parenteral applications

**DOI:** 10.3762/bjnano.11.16

**Published:** 2020-01-17

**Authors:** Eike Folker Busmann, Dailén García Martínez, Henrike Lucas, Karsten Mäder

**Affiliations:** 1 Institute of Pharmacy, Martin Luther University Halle-Wittenberg, Halle (Saale), Germany

**Keywords:** cellular toxicity, isotonicity, nanoemulsion, phase inversion, solvent free, surface properties

## Abstract

Lipid nanoemulsions are attractive drug delivery systems for lipophilic drugs. To produce nanoemulsions with droplets of very small diameter (<100 nm), we investigated thermotropic phase transitions as an alternative to the standard procedure of high-pressure homogenization. Employing shock dilution with ice-cold water during the phase inversion gives the opportunity to produce nanoemulsions without any use of potentially toxic organic solvents. The systematic investigation of the relation of the three involved components surfactant, aqueous phase and lipid phase showed that depending on the ratio of surfactant to lipid the emulsions contained particles of diameters between 16 and 175 nm with narrow polydispersity index distributions and uncharged surfaces. Nanoemulsions with particles of 50 and 100 nm in diameter showed very little toxicity to fibroblast cells in vitro. An unusual, exponential-like nonlinear increase in osmolality was observed with increasing concentration of the nonionic surfactant Kolliphor HS 15. The experimental results indicate, that nanoemulsions with particles of small and tunable size can be easily formed without homogenization by thermal cycling.

## Introduction

Nanoscaled drug delivery systems such as solid lipid or polymeric nanoparticles, nanocapsules, liquid nanoemulsions, liposomes and micelles can be used to carry poorly water soluble ingredients of pharmaceuticals for parenteral applications [1–3]. Thereby, the physical entrapment of the active ingredients into the core of the nanoparticles gives the possibility to solubilize and protect the sensitive drugs or contrast agents [2,4–5]. Their pharmacokinetics, including the distribution from the blood stream into the tissue, depend mainly on the size and shape, the surface composition, the charge as well as the flexibility of the nanoparticles [6–9]. Shock dilution with ice-cold water during phase inversion of the emulsion gives the opportunity to produce nanocapsules without the use of any potentially toxic organic solvent at low energy cost [10–11]. The choice and the amount of the surfactant show different effects on the final product. For example, these factors influence the feasibility of forming stable nanostructures, they affect the phase inversion zone, the in vitro cellular toxicity and the above mentioned particle and surface properties [3–4,12].

There are marketed drug products with significant hyperosmolality up to 2000 mOsmol/kg [13]. However, parenteral products should aim at isotonicity to reduce the risk of possible crenation (shriveling) or hemolysis of the red blood cells as well as significant pain at the site of injection or even phlebitis [13–14]. Therefore, a balance of increasing osmolality and the concentration of the dissolved educts needs to be considered to achieve isotonic nanoemulsions ensuring a painless intravenous application. The osmolality *O*(*c*) increases usually linearly with the solute concentration *c*, depending on the osmotic coefficient ϕ, the number of ions or particles *n* and the molecular weight *M* [15]:

[1] $$O\left(c\right)=\frac{\phi n}{M}\cdot c$$

However, the osmolality of many surfactants approaches a plateau above the critical micelle concentration. The formation of a separate micellar phase leads to a constant monomer concentration, therefore to a thermodynamically constrained system with a constant chemical potential and hence a constant osmotic pressure [16].

The aim of this study was to investigate the phase inversion-based production of a lipid nanocarrier without using phospholipids. Thus, instead of solid shelled nanocapsules, flexible nanoemulsions should be formed. Therefore, we systematically investigated the impact of the sodium chloride concentration on the phase inversion and the influence of the three involved components surfactant, aqueous phase and lipid phase on the formation of stable isotonic nanoemulsions. Furthermore, the relation of the particle composition, the particle size and the in vitro toxicity to fibroblasts was investigated.

## Results and Discussion

### Influence of the salinity on the phase inversion and the formation of nanoemulsions

**Figure 1:**
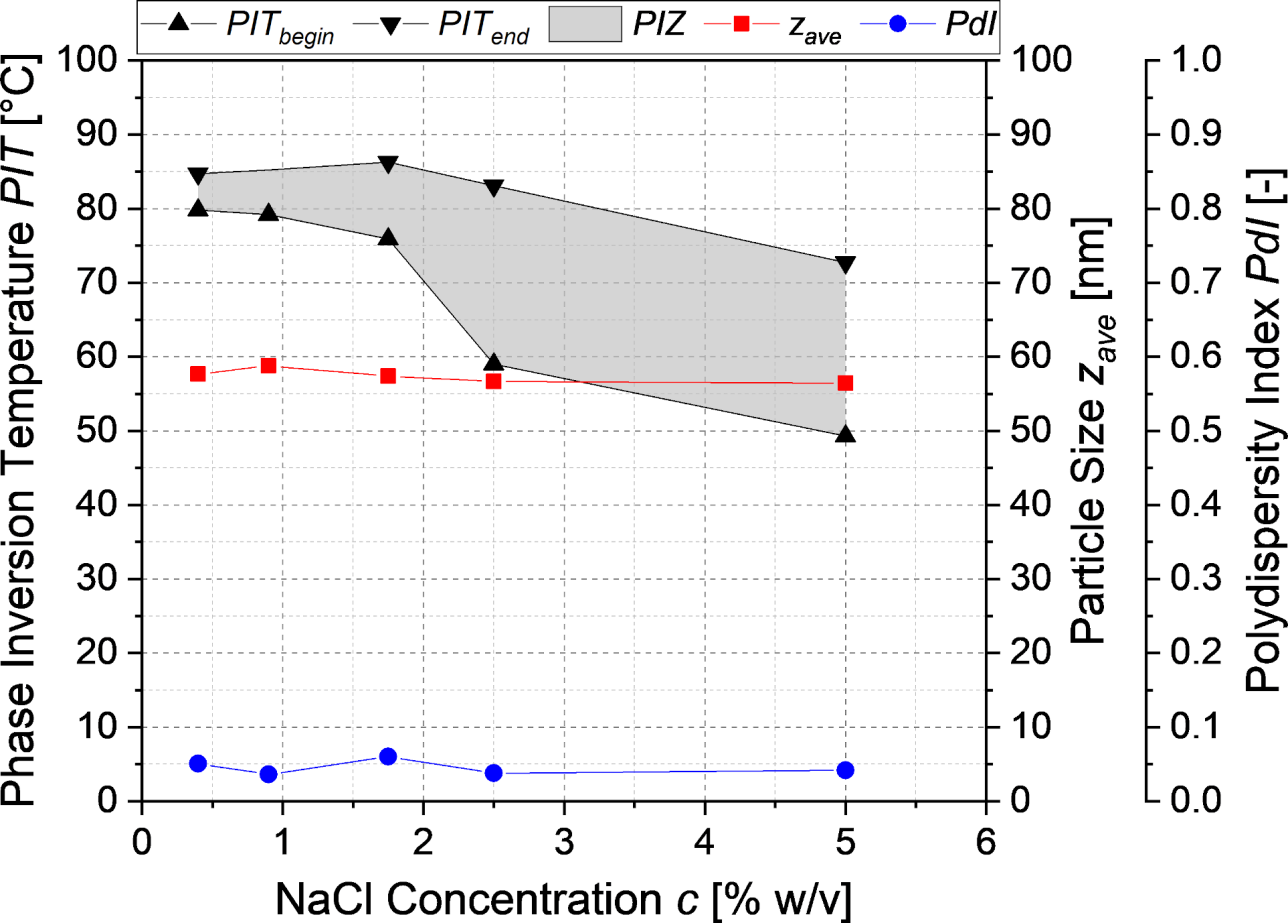
Influence of the salinity of the aqueous phase on the phase inverson zone (PIZ) for the nanoemulsions at a composition of MCT/Kolliphor/NaCl solution (20:20:60) along with the nanoparticle diameter and the PDI of the resulting nanoemulsion.

To investigate the influence of the salinity of the aqueous phase on the incipient phase inversion and hence on the point of forming stable but nonisotonic nanoemulsions, the conductivity of a medium chain triglyceride (MCT)/Kolliphor^®^ HS 15/NaCl solution (20:20:60) was measured as shown in Figure 1. Increasing the salinity of the aqueous phase resulted in a significant decrease of the temperature at the incipient phase inversion from 81.5 °C at 0.4 wt % NaCl concentration to 65.4 °C at 5 wt % NaCl concentration. The particles in the nanoemulsion had an average diameter (*z*_ave_) of 56–59 nm and a remarkably narrow polydispersity index (PDI) distribution of 0.03–0.06. The salinity did not have a significant influence on the particle diameter and the PDI of the nanoemulsion formed by shock dilution.

**Figure 2:**
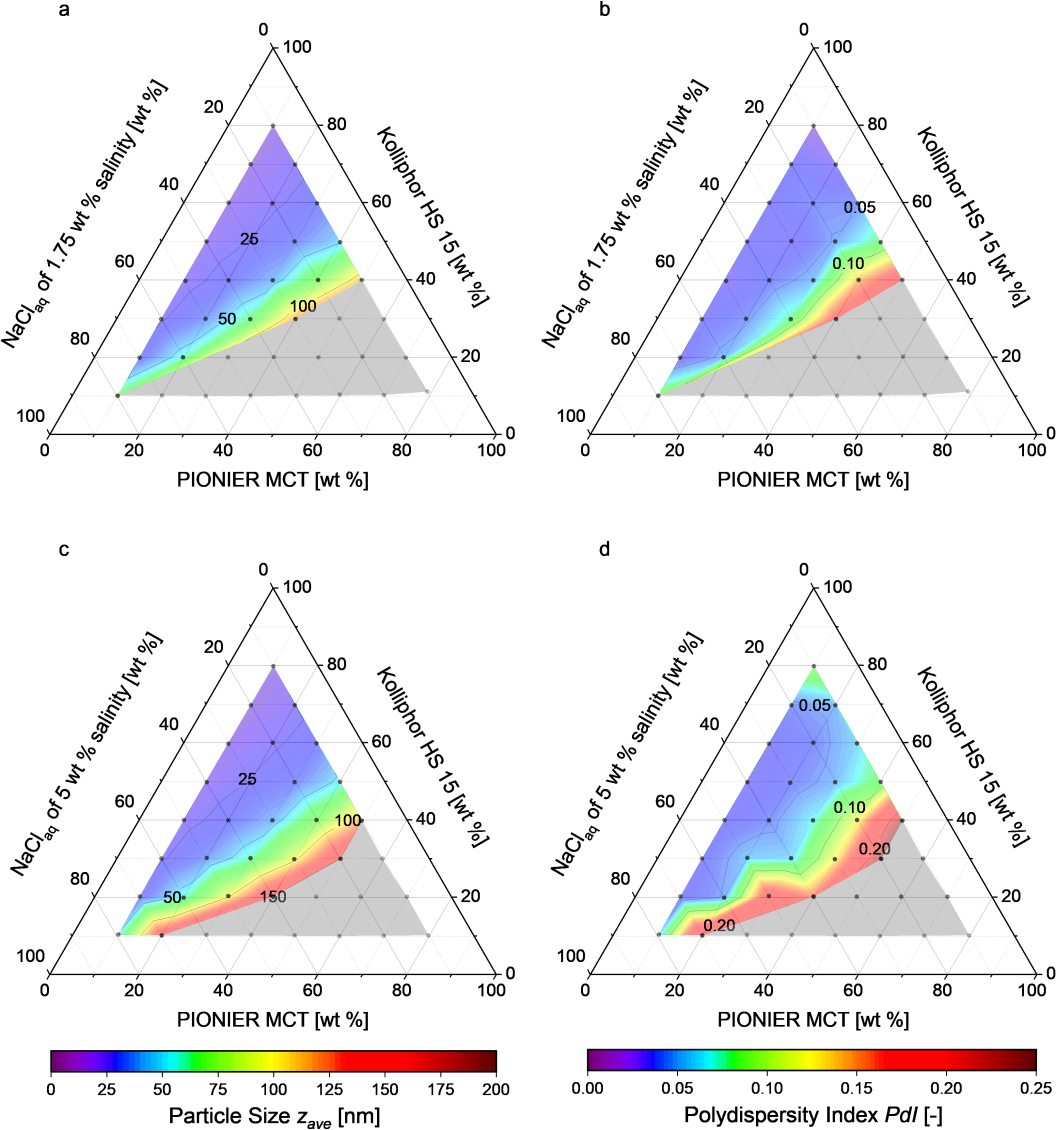
Ternary phase diagrams giving the particle diameter and the PDI of the nonisotonic nanoemulsions produced in (a, b) 1.75 wt % and (c, d) 5 wt % NaCl aqueous solution. Each dot represents the composition of the solution prepared for shock dilution. Formation of stable nanoemulsions occurs in the colored zone. Creaming coalescence or no phase inversion were observed in the grey zone.

Figure 2 shows the particle diameters and the PDIs of different nanoemulsions prepared using 1.75 wt % and 5 wt % NaCl solutions. Nanoemulsions were successfully formed using solutions of the compositions indicated by the colored zones. The solutions corresponding to the grey zones did not yield any stable nanoemulsions because they (1) resulted in creaming and/or coalescence directly after the shock dilution, or (2) the phase inversion temperature was higher than the boiling point of the aqueous phase. For solutions of higher salinity the area in which nanoemulsions were formed is enlarged, because the phase inversion temperature lies further below the boiling point of the aqueous phase. This facilitated producing stable nanoemulsions with particles of 16–150 nm in diameter with narrow PDI distributions of 0.02–0.23. Increasing the mass share of the surfactant Kolliphor HS 15 led to smaller particles and narrower PDI distributions.

### Influence of the nonionic surfactant Kolliphor HS 15 on the osmolality

**Figure 3:**
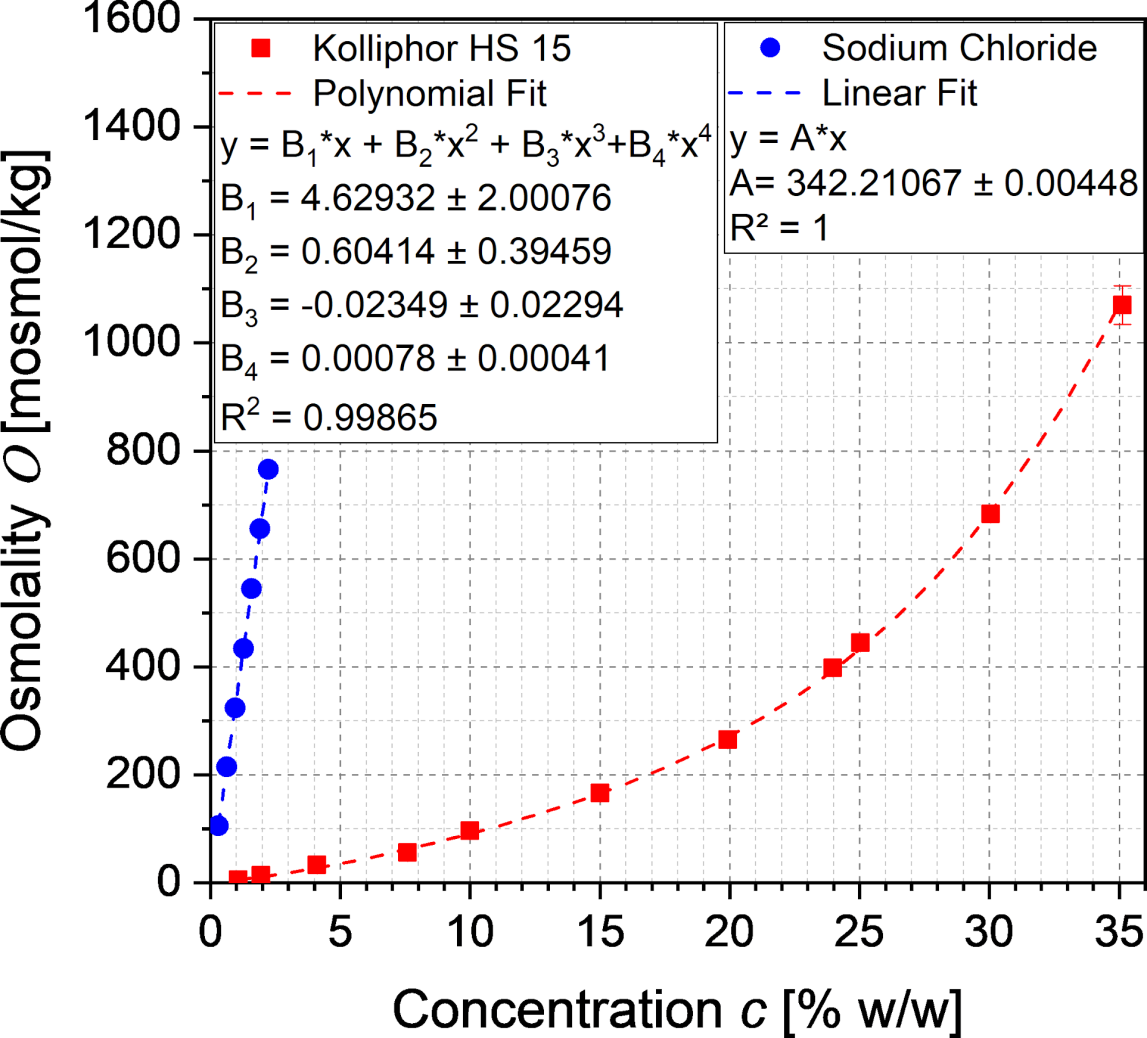
Ideal osmolality of aqueous sodium chloride solutions after Ph. Eur. 2.2.35 and experimental osmolality of aqueous dissolved Kolliphor HS 15, fitted according to Equation 1 and Equation 2.

The osmolality is an important parameter of the toxicity of parenteral dosage forms. The ideal osmolality values of an aqueous sodium chloride solution according to Ph. Eur. 2.2.35 and the experimentally determined osmolality of aqueous Kolliphor HS 15 are shown in Figure 3.

The linearly increasing osmolality of the aqueous solution of sodium chloride was fitted with the linear van’t Hoff law (Equation 1) at a coefficient of determination of 100%, and the reciprocal slope A was used for the term


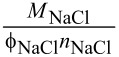


(see Equation 3) for the calculation of the salinity of isotonic nanoemulsions.

With increasing the concentration of the nonionic surfactant Kolliphor HS 15 the osmolality of the aqueous solution increased unexpectedly exponential-like instead of approaching the expected plateau above the critical micelle concentration. Intense literature research revealed only a single publication by Viegas and Henry [15] describing such a phenomenon for strongly interacting nonionic surfactants. The osmolality of solutions of such surfactants increases nonlinearly with the solute concentration due to increasing polymer–solvent hydrogen bonding at low temperatures, which prevents the flow of solvent molecules in the solution and thus affects osmosis. This phenomenon is described by a modified polynomial van’t Hoff equation of 4th degree introduced by Huggins and Flory with the association or interacting constants *b*1, *b*2 and *b*3:

[2] $$O\left(c\right)=\frac{\phi n}{M}\cdot c+\frac{\phi nb_{1}}{M}\cdot c^{2}+\frac{\phi nb_{2}}{M}\cdot c^{3}+\frac{\phi nb_{3}}{M}\cdot c^{4}$$

The experimental data corresponded well to the modified polynomial van’t Hoff equation of 4th degree with a coefficient of determination of 99.865%. Thus, the equation was used for the calculation of the osmolality *O*_Kol_(*c*_Kol,p_).

### Preparation of isotonic nanoemulsions

Given an osmolality of whole blood of 302 ± 5 mOsmol/kg and of blood plasma of 291 ± 4 mOsmol/kg [17], it was decided to produce nanoemulsions with a target osmolality of 300 mOsmol/kg. The necessary salinity of the aqueous phase for shock dilution was calculated according to Equations 8 to 11 for a product MCT mass share *x*_MCT,p_ of 8 wt %, which resulted in solutions of varying NaCl concentration (see Figure S1 in Supporting Information File 1).

**Figure 4:**
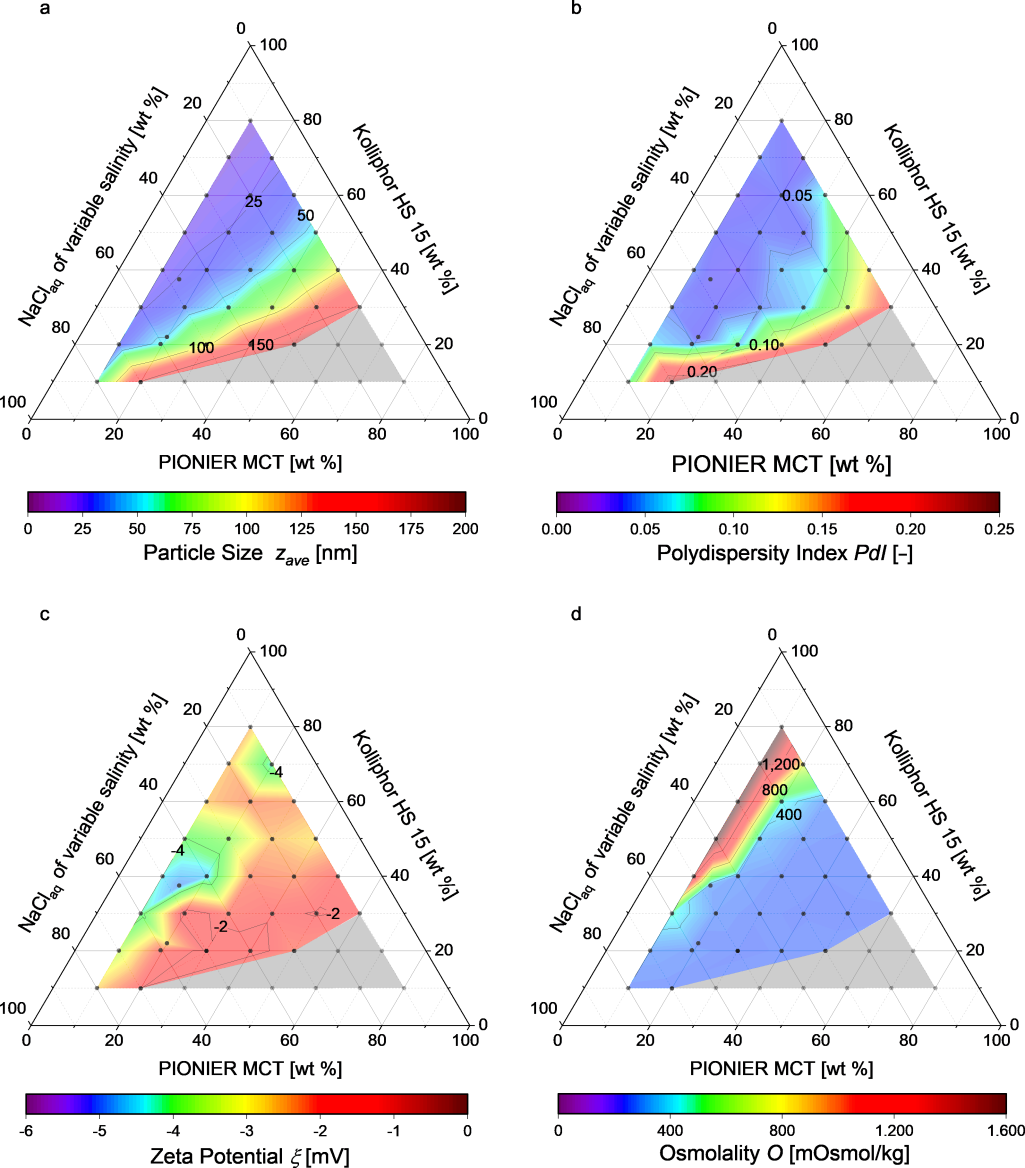
Ternary phase diagrams showing (a) the resulting particle diameter, (b) the PDI, (c) the zeta potential and (d) the osmolality. Each dot represents a certain solution composition. The formation of stable nanoemulsions occurs in the colored zone, while creaming coalescence or no phase inversion were observed in the grey zone.

Using this equation system for the production of isotonic nanoemulsions led to high salinities of up to 25 wt % in the aqueous phase before shock dilution. Increasing the salinity of the aqueous phase decreased the phase inversion temperature. In ternary phase diagrams, this decrease of the phase inversion temperature enlarged the area in which stable nanoemulsions were formed compared to the nonisotonic nanoemulsions produced in aqueous solutions of 1.75 wt % and 5 wt % NaCl concentration, as shown in Figure 4.

**Figure 5:**
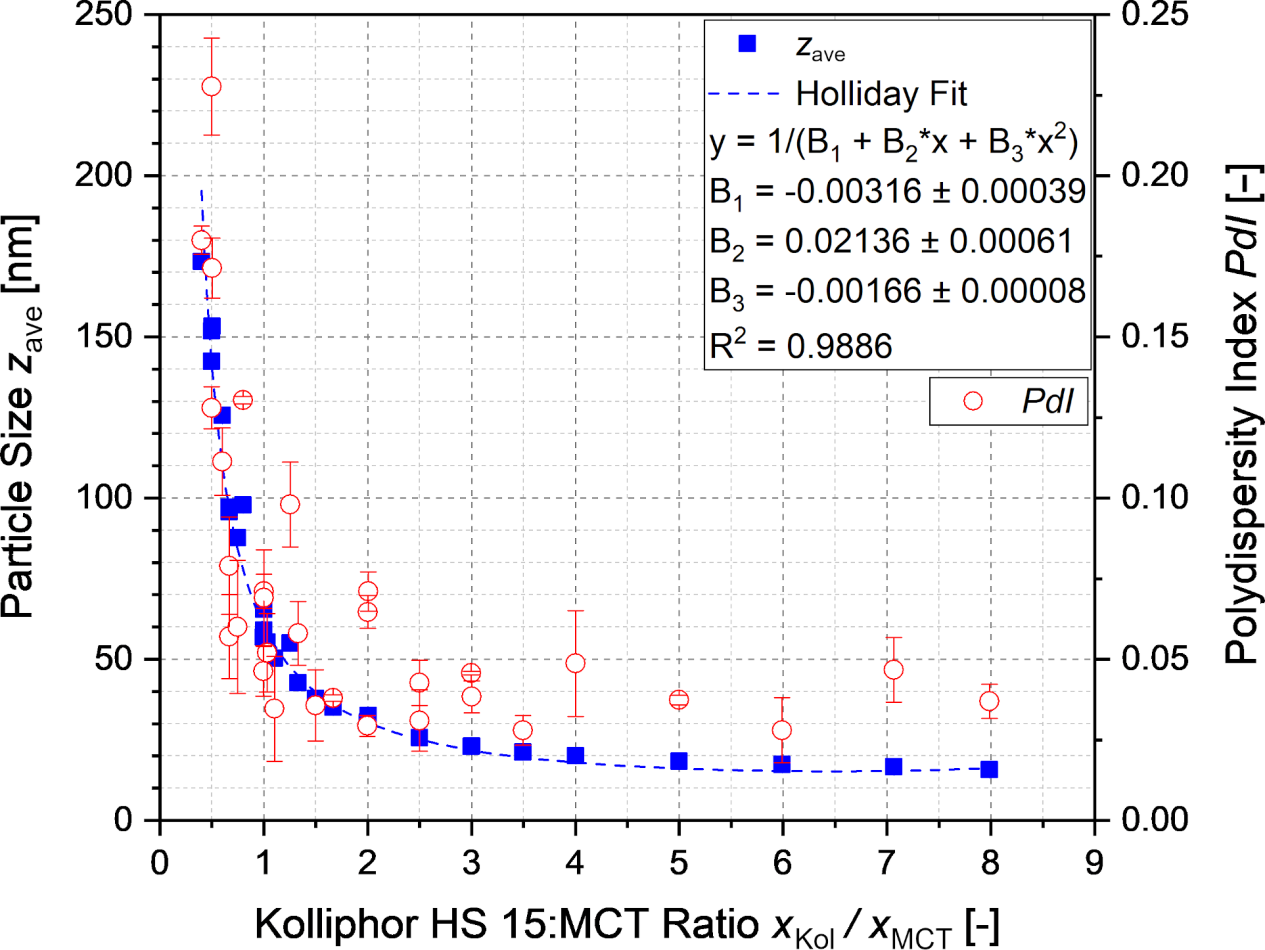
Particle diameters and PDI of the nanoemulsions depending on the Kolliphor HS 15:MCT ratio.

Nanoemulsions with particles of 16–175 nm in diameter were formed with narrow PDI distributions of 0.02–0.25, as illustrated in Figure 4a and Figure 4b. All nanoemulsions were stable over one month at room temperature. The sizes of the particles within the nanoemulsions depended only on the ratio of Kolliphor HS 15 to MCT. The dependence depicted in Figure 5 agrees well with a Holliday fit yielding a coefficient of determination of 98.86%. The increase of the concentration of the surfactant Kolliphor HS 15 and hence the increase of the ratio of Kolliphor HS 15 to MCT resulted in a prompt decrease of the particle diameter. The constant limit is reached at ratios above three.

The zeta potential of all nanoemulsions shown in Figure 4c was slightly negative between −1.6 and −4.6 mV, measured in 0.1× PBS at physiological pH 7.4. Thus, the composition of the nanoemulsion had no clear influence on the surface charge. Furthermore, isotonicity was nearly achieved for the formulations in the blue zone of Figure 4d. Formulations shown in the green, yellow and red zones resulted in hypertonicity although they were prepared in an NaCl free aqueous phase (compare Figure S1 in Supporting Information File 1) due to a final Kolliphor HS 15 concentration above 21 wt %. In these zones, isotonicity may be achieved by producing nanoemulsions using a larger amount of aqueous solution for the shock dilution, which would also lead to a smaller MCT mass share in the product nanoemulsion.

### Stability of the isotonic nanoemulsions

**Figure 6:**
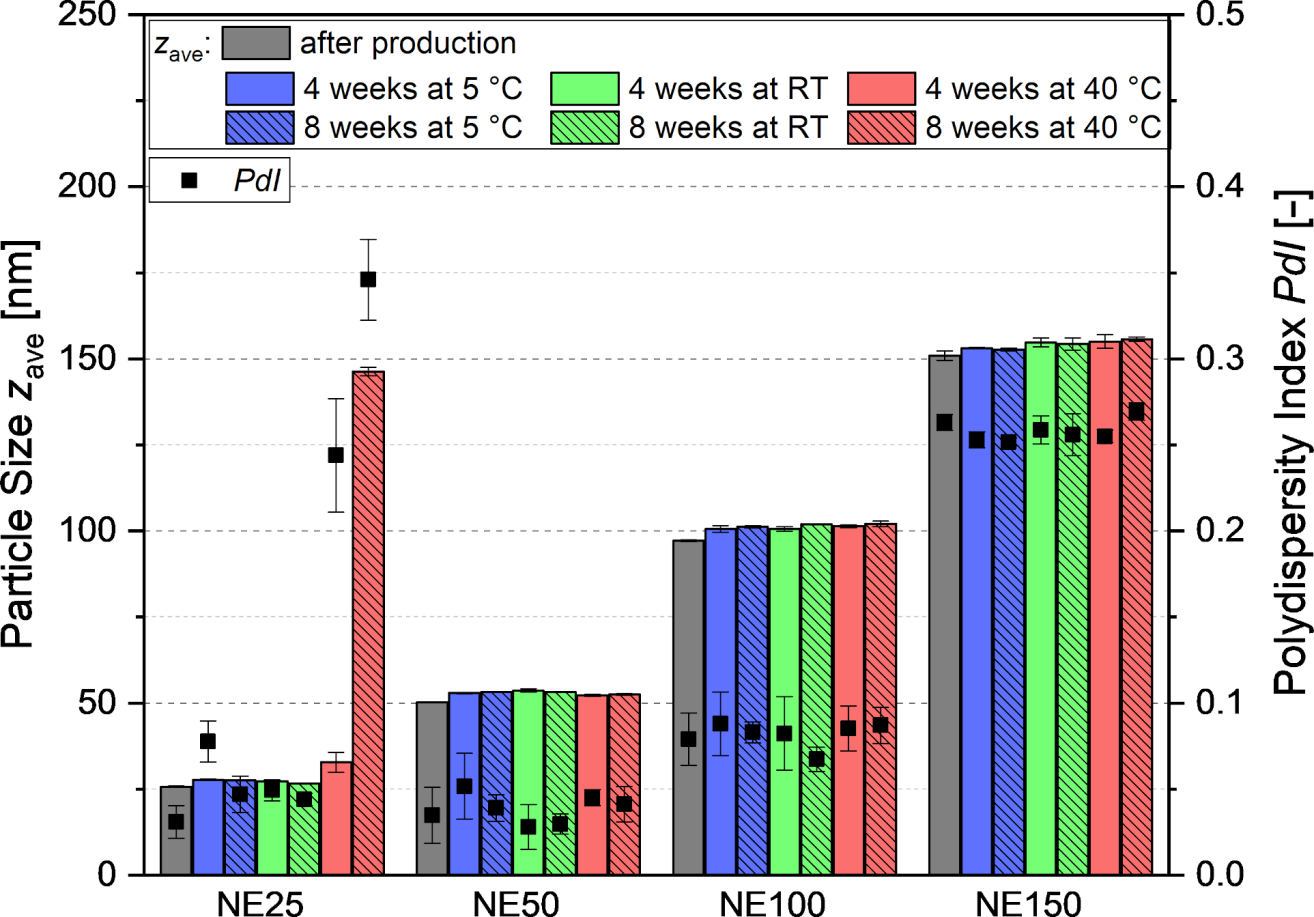
Long-term stability of four selected nanoemulsions with particles of approximately 25 (NE25), 50 (NE50), 100 (NE100) and 150 nm (NE150) in diameter at the recommended storage conditions according to the ICH guidelines Q1A of 5 ± 3 °C, room temperature (RT) and 40 ± 2 °C.

To investigate the stability of the nanoemulsions, four emulsions with particles of approximately 25 (NE25), 50 (NE50), 100 (NE100) and 150 nm (NE150) in diameter were chosen. Their composition and the final particle diameters observed in nine independently produced batches are listed in Table 1. Four selected nanoemulsions were stored at 5 ± 3 °C, room temperature (RT) and 40 ± 2 °C according to the ICH Guidelines Q1A. The impact of the storage conditions on the particle diameter is illustrated in Figure 6. With the exception of emulsion NE25, all nanoemulsions were stable over the course of eight weeks at the three storage conditions. Only NE25 underwent creaming and coalescence at 40 °C resulting in a significant increase of the particle diameter up to 146 nm and a high PDI of 0.35 within 8 weeks of storage. Hence, it is strongly recommended to store the nanoemulsions with particles of very small size at chilled conditions.

**Table 1:** Composition of the nanoemulsions NE25, NE50, NE100 and NE150 after shock dilution along with the particle diameters and PDI values of nine independently produced batches, *c*_NaCl_ describes the salinity of the aqueous phase of the final product.

	NE25	NE50	NE100	NE150

MCT [wt %]	8	8	8	8
Kolliphor HS 15 [wt %]	20	8.8	5.33	4
NaCl solution [wt %] at *c*_NaCl_ [wt %]	72 0.084	83.2 0.654	86.67 0.763	88 0.798
particle diameter [nm]	26.2 ± 0.3	51.4 ± 0.7	99.4 ± 2.3	145.9 ± 4.7
polydispersity index [–]	0.046 ± 0.014	0.038 ± 0.006	0.092 ± 0.021	0.251 ± 0.091

### Cellular toxicity of the isotonic nanoemulsions

**Figure 7:**
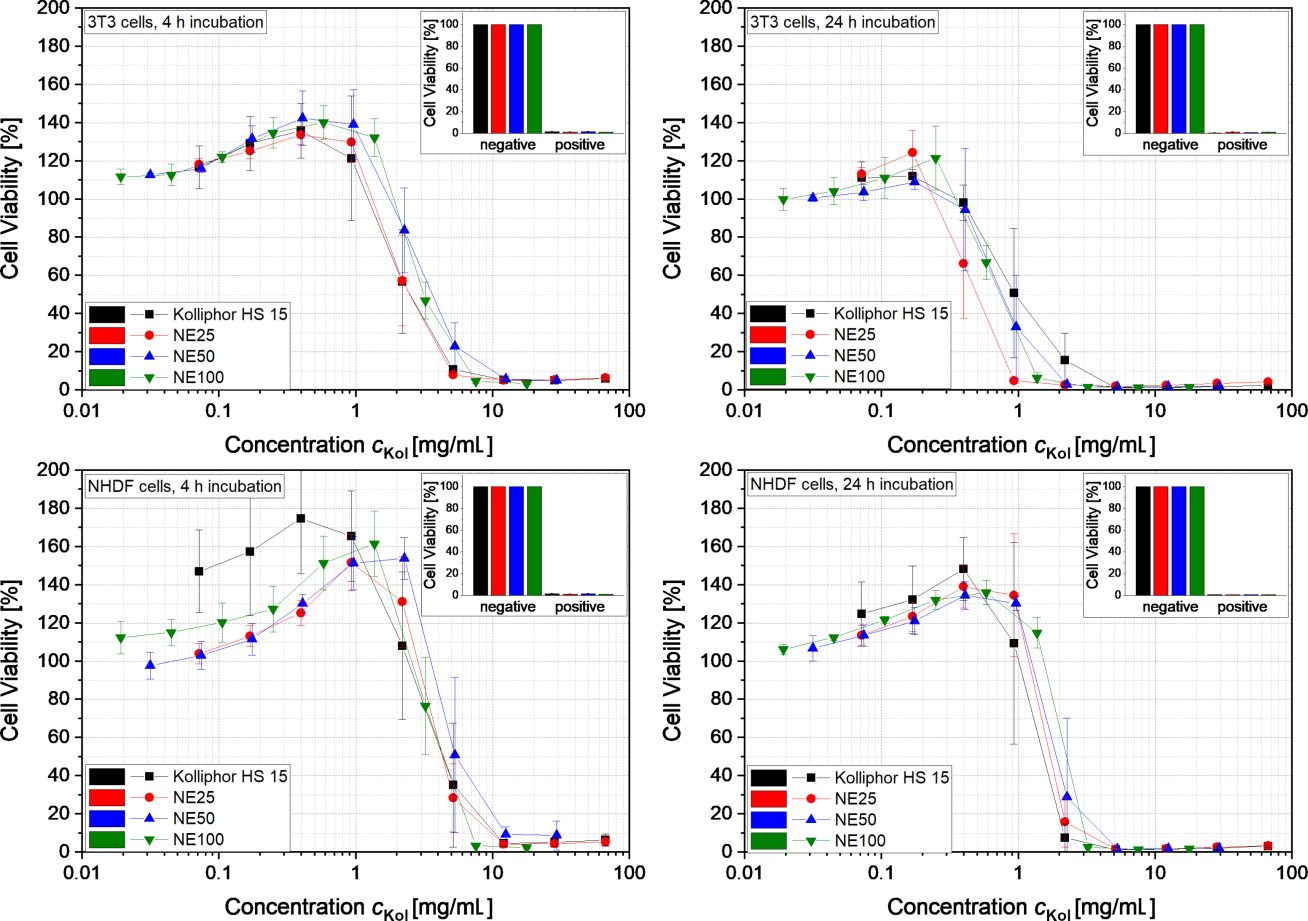
Viability of the cells of lines 3T3 and NHDF as a function of the Kolliphor HS 15 concentration *c*_Kol_ in the nanoemulsions NE25, NE50 and NE100 as well as the aqueous Kolliphor HS 15 solution (*n* = 3).

The cellular toxicity of the nanoemulsions NE25, NE50 and NE100 as well as the aqueous Kolliphor HS 15 solution to normal human dermal fibroblasts (NHDF) and mouse embryonic fibroblasts (3T3) was investigated. The impact of the nanoemulsions and the surfactant solution on the cell viability of the two cell lines after 4 and 24 h is illustrated in Figure 7. The viability of the cells treated with the different nanoemulsions is depicted as a function of the concentration of the surfactant Kolliphor HS 15. The resulting graphs indicate a similar behavior of all three nanoemulsions and the aqueous Kolliphor HS 15 solution, namely a decrease of the cell viability at a certain concentration *c*_Kol_. The 3T3 cells responded more sensitively to the nanoemulsions and the pure surfactant solution. Furthermore, the long incubation time of 24 h led to cell toxicity at slightly lower concentrations for both cell lines. For comparison, for the current nanoemulsions, the cell viability began to decrease at values of the Kolliphor HS 15 concentration about 5 to 10 times higher than observed for a similar system with nanocapsules containing phospholipids as shells and Solutol HS 15 (also called Kolliphor HS 15) as surfactant which were tested on HaCaT cells [4]. At a concentration slightly lower than the inhibiting Kolliphor HS 15 concentration, an increased cell viability was observed, which might be caused by a stimulated metabolism of the cells.

Table 2 lists the mean inhibitory concentration (IC_50_) of Kolliphor HS 15 in an aqueous solution of Kolliphor HS 15 (second column) and the IC_50_ values of Kolliphor HS 15 in the nanoemulsions NE25, NE50 and NE100 (third, fourth and fifth column). The values in brackets refer to the concentration of MCT + Kolliphor HS15 in the nanoemulsions NE25, NE50 and NE100. The corresponding cell viability graphs are shown in Figure S2 in Supporting Information File 1. The IC_50_ values of Kolliphor HS 15 in the different nanoemulsions and the pure surfactant solution were similar for the different cell lines and incubation times employed. Thus, mainly the surfactant Kolliphor HS 15 inhibited cell viability at high concentration, since all remaining components are considered nontoxic. The IC_50_ values of the nanoemulsions (MCT + Kolliphor HS15) increased slightly with increasing particle diameter due to the lower Kolliphor HS 15 concentration in the nanoemulsions containing larger nanoparticles.

**Table 2:** Mean inhibitory concentration (IC_50_ in mg/mL) of Kolliphor HS 15 in an aqueous solution of Kolliphor HS 15 and IC_50_ of Kolliphor HS 15 in the nanoemulsions NE25, NE50 and NE100. The values in brackets refer to the concentration of MCT + Kolliphor HS15 in NE25, NE50 and NE100.

IC_50_ in mg/mL of:	Kolliphor HS 15	NE25	NE50	NE100

3T3, 4 h incubation	2.7 ± 0.9	2.6 ± 0.8 (3.6 ± 1.1)	3.8 ± 0.9 (7.3 ± 1.7)	3.3 ± 0.5 (8.2 ± 1.2)
NHDF, 4 h incubation	4.9 ± 2.2	4.5 ± 0.6 (6.3 ± 0.8)	6.2 ± 2.5 (11.9 ± 4.8)	4.6 ± 0.9 (11.5 ± 2.2)
3T3, 24 h incubation	1.2 ± 0.5	0.5 ± 0.2 (0.7 ± 0.2)	0.8 ± 0.3 (1.6 ± 0.6)	0.8 ± 0.1 (2.0 ± 0.2)
NHDF, 24 h incubation	1.5 ± 0.4	1.8 ± 0.2 (2.5 ± 0.3)	2.3 ± 0.9 (4.4 ± 1.7)	2.4 ± 0.1 (6.1 ± 0.2)

**Figure 8:**
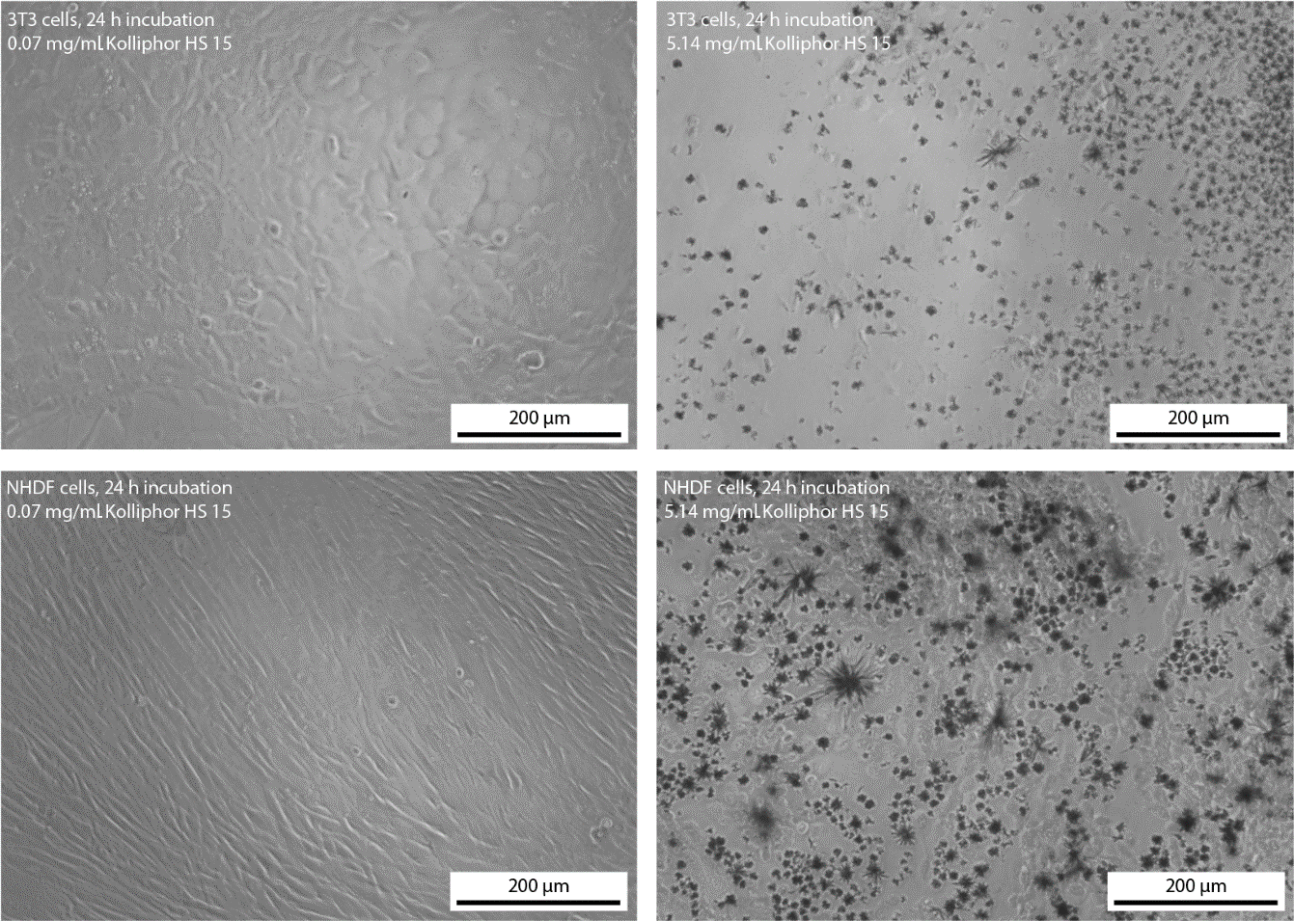
Morphology of the 3T3 and NHDF cells after 24 h incubation ion solutions with a concentration of the surfactant Kolliphor HS 15 of 0.07 and 5.14 mg/mL.

Figure 8 shows the morphology of the 3T3 and NHDF cells incubated for 24 h in a nontoxic solution with a concentration of Kolliphor HS 15 of 0.07 mg/mL and a highly toxic solution with a concentration of Kolliphor HS 15 of 5.14 mg/mL. The microscopic pictures confirm the toxicity of the surfactant Kolliphor HS 15 at high concentration. The cells did not show any change in their morphology when dissolved in solutions of low surfactant concentration. However, the cells showed a shriveled morphology at high surfactant concentration. Furthermore, the formation of crystals was observed, which might consist of poorly water soluble 12-hydroxystearic acid as a metabolic degradation product of Kolliphor HS 15 at toxic and high surfactant concentration. 12-Hydroxystearic acid is known to form needle like structures. The formation of these crystals of 12-hydroxystearic acid might have caused the cell death in vitro for both cell lines. We assume, that the formation of 12-hydroxystearic acid-based precipitates is unlikely to occur in vivo because of the different transport and metabolic conditions.

## Conclusion

The experimental results indicate that nanoemulsions with small particles of tunable size can be easily formed without homogenization by thermal cycling. Solutions of the nonionic surfactant Kolliphor HS 15 showed a nonlinear increase of osmolality with increasing Kolliphor HS 15 concentration, which corresponds well to the modified polynomial van’t Hoff equation of 4th degree introduced by Huggins and Flory. This led to the hypertonicity of solutions with Kolliphor HS 15 concentration higher than 21 wt %. Considering this effect when calculating the salinity of the aqueous phase, stable isotonic and phospholipid-free MCT nanoemulsions were successfully produced with particles of tunable diameters between 16 to 175 nm and narrow PDI distributions depending on their lipid:surfactant ratio. Using a nonionic surfactant resulted in an uncharged surface of the emulsion droplets. The nanoemulsion with small particles of 25 nm in diameter showed an slightly increased cytotoxicity in comparison to the barely toxic nanoemulsions with particles of 50 and 100 nm in diameter. This effect is mainly caused by the very high amount of Kolliphor HS 15 in the nanoemulsion with the smallest particles. By choosing nanoemulsions with particles larger than 50 nm in diameter or by dilution of the nanoemulsions containing smaller particles with ice-cold water, very high surfactant concentrations could be avoided and the resulting MCT nanoemulsions might be suitable as potential drug delivery systems for intravenous applications. The presented phase inversion-based process offers a suitable alternative to the production of nanoemulsions based on high pressure homogenization. Particularly, nanoemulsions of particles with diameters smaller than 100 nm can be produced with remarkably narrow PDI distributions while reducing the demand of equipment, the process expenditure as well as the production volume.

## Experimental

### Materials

PIONIER MCT (medium chain triglyceride) was provided by Hansen & Rosenthal KG (Hamburg, Germany). Kolliphor HS 15 (macrogol 15 hydroxystearate) was provided by BASF SE (Ludwigshafen, Germany). Sodium chloride was purchased from Grüssing GmbH (Filsum, Germany), the components for the cell culture medium Dulbecco’s Modified Eagle Medium – high glucose (DMEM), fetal calf serum (FCS), penicillin-streptomycin, ʟ-glutamine solution and sodium pyruvate solution as well as the fluorescent dye resazurin sodium salt were purchased from Sigma-Aldrich Chemie GmbH (Germany, Steinheim). The near infrared fluorescent dye DiR was purchased from Invitrogen/Thermo Fisher Scientific Inc. (Carlsbad, USA). 0.2 µm sterile filtered water was used in all experiments and analytics and was double distilled.

### Preparation of nanoemulsions

**Figure 9:**
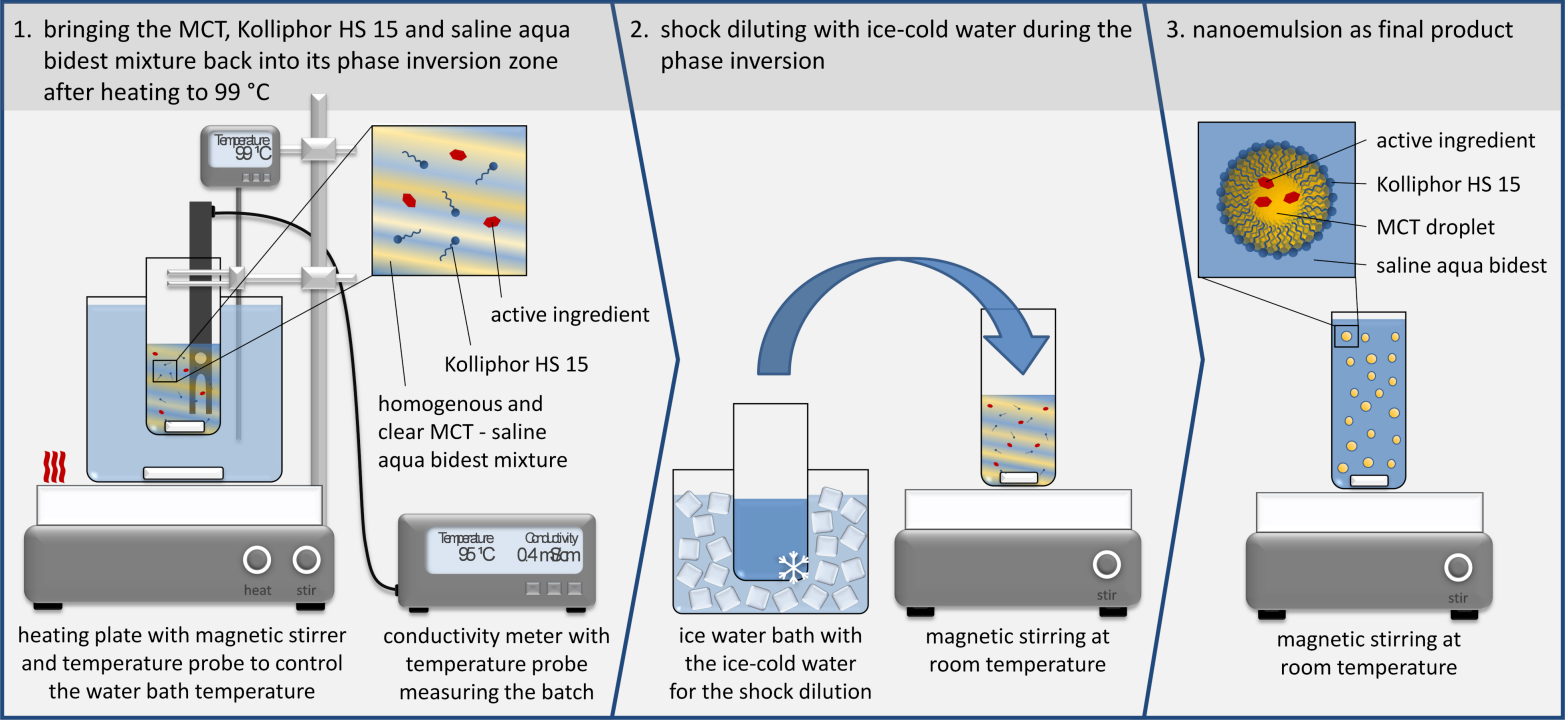
Scheme of the experimental set-up and the method of phase inversion-based production of the nanoemulsions.

All nanoemulsions were prepared by a modified phase inversion-based process, which was initially developed by Heurtault et al. [10]. For the encapsulation of the fluorescent dye DiR as a potential label for noninvasive optical in vivo imaging, the solvent ethanol was evaporated from the dye stock solution and the remaining DiR was dissolved in MCT at a concentration of 0.3 mg/g. Kolliphor HS 15, which was molten at 50 °C, and MCT were dispersed in aqueous NaCl solution under magnetic stirring at ≈750 rpm. The emulsion was heated to 99 °C undergoing a phase inversion from an o/w to a w/o emulsion. The emulsion then was cooled back into its phase inversion zone, in which the mixtures turned transparent, and shock diluted with ice-cold water, as shown in Figure 9.

The amount of the respective educts was calculated with the following equations. The mass of MCT *m*_MCT_ is the product of its desired mass share *x*_MCT,p_ in the nanoemulsion after shock dilution, which was set to 8 wt % in this study, and the desired total amount of the nanoemulsion *m*_tot_:

[4] $$m_{\text{MCT}}=m_{\text{tot}}\cdot x_{\text{MCT},\text{p}}$$

The product of *m*_MCT_ and the desired ratio of the mass share of Kolliphor HS 15 *x*_Kol,0_ and MCT *x*_MCT,0_ results in the mass of Kolliphor HS 15 *m*_Kol_:

[5] $$m_{\text{Kol}}=m_{\text{MCT}}\cdot \frac{x_{\text{Kol},0}}{x_{\text{MCT},0}}=m_{\text{tot}}\cdot x_{\text{MCT},\text{p}}\cdot \frac{x_{\text{Kol},0}}{x_{\text{MCT},0}}$$

The mass of the aqueous NaCl solution *m*_NaCl–Sol_ is calculated by multiplying *m*_MCT_ and the ratio of *x*_Kol,0_ and the mass share of the NaCl solution *x*_NaCl,0_:

[6] $$\begin{array}{c} m_{\text{NaCl–Sol}}=m_{\text{MCT}}\cdot \frac{x_{\text{NaCl–Sol}\text{,0}}}{x_{\text{MCT}\text{,0}}}\\ =m_{\text{tot}}\cdot x_{\text{MCT},\text{p}}\cdot \frac{1-x_{\text{MCT},0}-x_{\text{Kol},0}}{x_{\text{MCT},0}} \end{array}$$

The mass of the ice-cold water *m*_ice water_ for shock dilution is the difference between the desired total mass of the nanoemulsion and the components calculated beforehand:

[7] $$\begin{array}{c} m_{\text{ice}\;\text{water}}=m_{\text{tot}}-m_{\text{MCT}}-m_{\text{Kol}}-m_{\text{NaCl–Sol}}\\ =m_{\text{tot}}-m_{\text{tot}}\cdot x_{\text{MCT},\text{p}}\\ \cdot \left(1+\frac{x_{\text{Kol},0}}{x_{\text{MCT},0}}+\frac{1-x_{\text{MCT},0}-x_{\text{Kol},0}}{x_{\text{MCT},0}}\right) \end{array}$$

To achieve isotonic nanoemulsions after shock dilution, the targeted osmolality of the saline aqueous phase *O*_NaCl–Sol,p_(*c*_NaCl,p_) is calculated as the difference of the osmolality of blood *O*_blood_ and the osmolality of the dissolved Kolliphor HS 15 *O*_Kol,p_(*c*_Kol,p_):

[8] $$O_{\text{NaCl–Sol},\text{p}}\left(c_{\text{NaCl}\text{,p}}\right)=O_{\text{blood}}-O_{\text{Kol}\text{,p}}\left(c_{\text{Kol}\text{,p}}\right)$$

Combining Equation 8 with Equation 1 and Equation 2 leads to the necessary sodium chloride concentration *c*_NaCl,p_ to achieve the osmolality of blood and hence isotonicity:

[3] $$c_{\text{NaCl}\text{,p}}=\left(O_{\text{blood}}-O_{\text{Kol}}\left(c_{\text{Kol}\text{,p}}\right)\right)\cdot \frac{M_{\text{NaCl}}}{\phi_{\text{NaCl}}n_{\text{NaCl}}}$$

Here, the concentration of Kolliphor HS 15 equals its mass share in the final product and is calculated by multiplying *x*_MCT,p_ and the desired ratio of *x*_Kol,0_ and *x*_MCT,0_:

[9] $$c_{\text{Kol}\text{,p}}=x_{\text{Kol}\text{,p}}=x_{\text{MCT}\text{,p}}\cdot \frac{x_{\text{Kol}\text{,0}}}{x_{\text{MCT}\text{,0}}}$$

Finally, the sodium chloride concentration *c*_NaCl,0_ before shock dilution is calculated by:

[10] $$c_{\text{NaCl}\text{,0}}=c_{\text{NaCl}\text{,p}}\cdot \frac{m_{\text{NaCl–Sol}}+m_{\text{ice}\;\text{water}}}{m_{\text{NaCl–Sol}}}$$

In case the calculated sodium chloride concentration exceeded 25 wt %, *c*_NaCl,0_ was set to 25 wt % and the residual salt was added to the ice-cold water at the following concentration:

[11] $$\begin{array}{c} c_{\text{NaCl}\text{,ice}\;\text{water}}=\frac{\left(m_{\text{NaCl–Sol}}+m_{\text{ice}\;\text{water}}\right)\cdot c_{\text{NaCl}\text{,p}}}{m_{\text{ice}\;\text{water}}}\\ -\frac{m_{\text{NaCl–Sol}}\cdot c_{\text{NaCl}\text{,0}}}{m_{\text{ice}\;\text{water}}} \end{array}$$

### Characterization of the phase inversion

The temperature of the phase inversion from an o/w to a w/o emulsion was determined by measuring the significant conductivity decrease during the heating process with the Mettler Toledo S230 SevenCompact conductivity meter.

### Characterization of the osmolality

The osmolality was determined in triplicate with the KNAUER Semi-Mikro Osmometer. The osmolality of the sodium chloride solution (according to Ph. Eur. 2.2.35) was fitted linearly, and the experimentally determined osmolality of the aqueous solutions of Kolliphor HS 15 with different concentration were fitted with a polynomial function of 4th degree using the program Origin 2018G both with the intersection of the *y*-axis fixed at 0.

### Particle size and zeta potential measurement

Particle diameters and zeta potentials were determined with the Malvern Instruments Zetasizer Nano ZS. To determine the particle diameters, the samples were diluted 1:100 in water and three measurements of 15 runs were conducted at 25 °C in the backscattering mode. The zeta potential was determined in triplicate with samples diluted 1:10 in 0.1× PBS with pH 7.4 at 25 °C with 10 to 50 runs per measurement.

### Investigation of the stability

The samples were stored at 5 ± 3 °C, room temperature and at 40 ± 2 °C according to the storage conditions of the ICH guidelines Q1A.

### Toxicity on NHDF and 3T3 cells

Approximately 20,000 NHDF cells and 10,000 3T3 cells were seeded in 96 well plates and grown for 24 h at 37 °C and 5% CO_2_ in 100 µL of the corresponding cell culture media shown in Table 3. After adding 50 µL aseptic and 0.2 µm of the sterile, filtered and differently diluted nanoemulsions, the cells were incubated for 4 or 24 h. The cell viability was determined by a resazurin reduction assay. Therefore, 30 µL of a 0.15 mg/mL resazurin solution was added and the mixture was incubated for 2 h. Then, the fluorescence intensity was determines with the Cytation^TM^ 5 imaging reader (BioTek Instruments) using the RFP 531(excitation)/593(emission) filter set. The cell viability was expressed as a percentage of the negative controls (untreated cells) after subtraction of the blank. All experiments were conducted in triplicate. The mean inhibitory concentration IC_50_ was determined by linear interpolation.

**Table 3:** Composition of the cell culture media.

compounds in vol %	3T3 cell medium	NHDF cell medium

DMEM	86.80	87.63
FCS	9.55	9.64
penicillin-streptomycin	0.95	0.96
ʟ-glutamine solution	1.75	1.77
sodium pyruvat solution	0.95	–

## Supporting Information

File 1 Additional figures.
