# Supplementary material for: Phase inversion-based nanoemulsions of medium chain triglyceride as potential drug delivery system for parenteral applications
Source: Beilstein J Nanotechnol. 2020 Jan 17;11:213–24. doi: 10.3762/bjnano.11.16 (PMC7006485; doi:10.3762/bjnano.11.16)
Supplement: File 1 — Additional figures. [file Beilstein_J_Nanotechnol-11-213-s001.pdf]

BEILSTEIN JOURNAL OF NANOTECHNOLOGY

Supporting Information

for

Phase inversion-based nanoemulsions of medium chain triglyceride as potential drug delivery system for parenteral applications

Eike Folker Busmann, Dailén García Martínez, Henrike Lucas and Karsten Mäder

Beilstein J. Nanotechnol. **2020,** *11,* 213–224. doi:10.3762/bjnano.11.16

Additional figures

License and Terms: This is a supporting information file under the terms of the Creative Commons Attribution License (https://creativecommons.org/licenses/by/4.0). Please note that the reuse, redistribution and reproduction in particular requires that the authors and source are credited.
The license is subject to the *Beilstein Journal of Nanotechnology* terms and conditions: (https://www.beilstein-journals.org/bjnano)

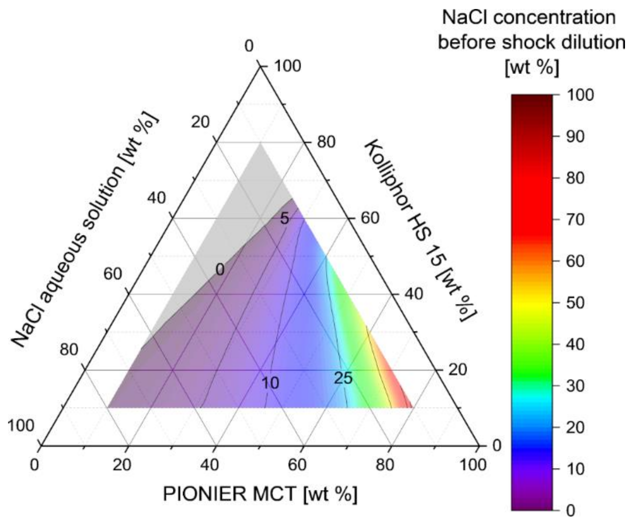

Figure S1: Ternary phase diagram showing the calculated sodium chloride concentration of the aqueous phase for the production of isotonic nanoemulsions according to Equation 10. The grey area resembles solutions for which the calculated salt concentration was negative.

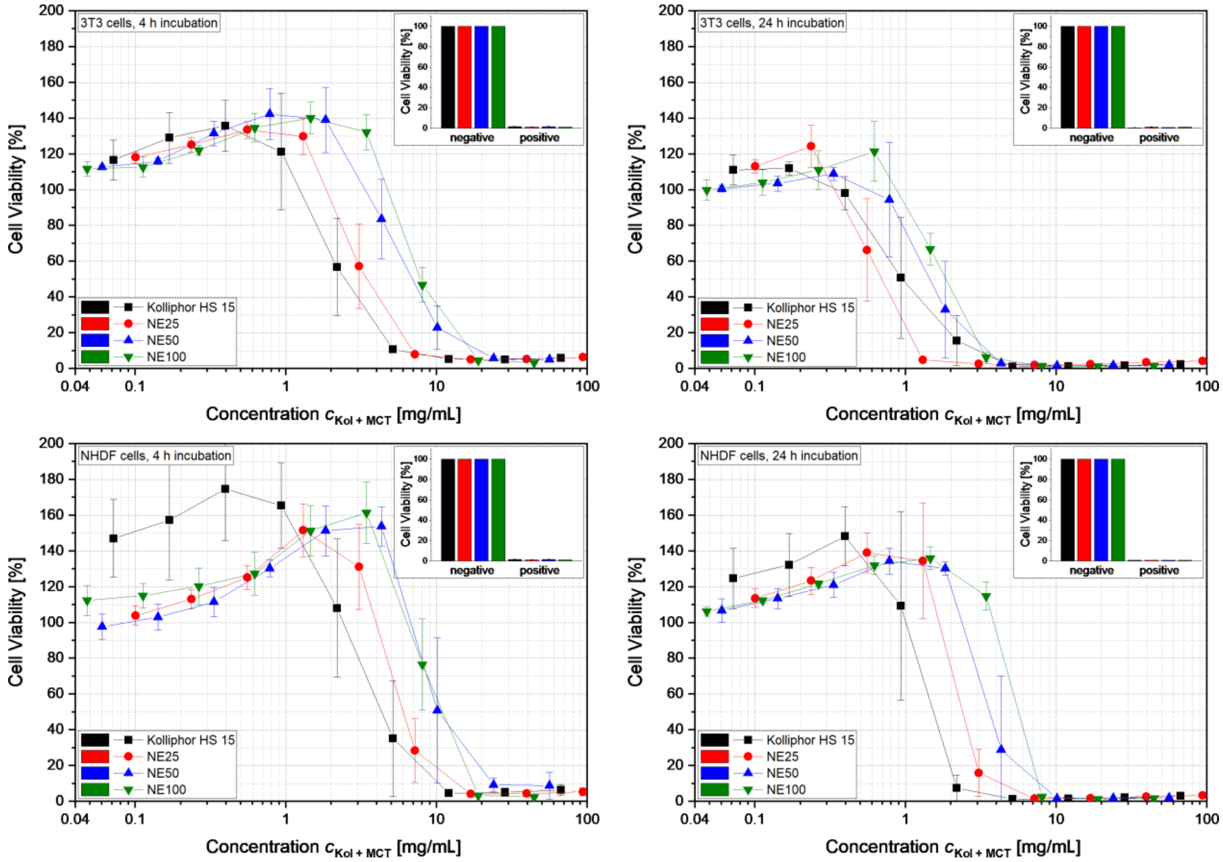

Figure S2: Viability of the cell of lines 3T3 and NHDF as a function of the concentration (Kolliphor HS 15 + MCT) of the nanoemulsions NE25, NE50 and NE100 as well as the concentration of an aqueous Kolliphor HS 15 solution (n = 3).
